# Asymmetric dimethylarginine and citrulline are risk factors for cardiovascular disease independently of both estimated and measured GFR

**DOI:** 10.1093/ckj/sfaf181

**Published:** 2025-06-13

**Authors:** Nikoline B Rinde, Toralf Melsom, Ole Martin Fuskevåg, Bjørn O Eriksen, Jon Viljar Norvik

**Affiliations:** Metabolic and Renal Research Group, UiT The Arctic University of Norway, Tromsø, Norway; Section of Nephrology, University Hospital of North Norway, Tromsø, Norway; Metabolic and Renal Research Group, UiT The Arctic University of Norway, Tromsø, Norway; Section of Nephrology, University Hospital of North Norway, Tromsø, Norway; Division of Diagnostic Services, Department of Laboratory Medicine, University Hospital of North Norway, Tromsø, Norway; Department of Clinical Medicine, UiT The Arctic University of Norway, Tromsø, Norway; Metabolic and Renal Research Group, UiT The Arctic University of Norway, Tromsø, Norway; Section of Nephrology, University Hospital of North Norway, Tromsø, Norway; Metabolic and Renal Research Group, UiT The Arctic University of Norway, Tromsø, Norway; Section of Nephrology, University Hospital of North Norway, Tromsø, Norway

**Keywords:** ADMA, cardiovascular disease, citrulline, glomerular filtration rate, SDMA

## Abstract

**Background:**

Nitric oxide (NO) is crucial for endothelial dysfunction and its deficiency is linked to cardiovascular disease (CVD) and impaired kidney function. While research has explored NO metabolism in individuals with kidney disease, diabetes mellitus (DM) or CVD, the relationship in healthy individuals remains unclear. Studies using estimated glomerular filtration rate (eGFR) for kidney function may introduce non-GFR-related factors, confounding the results. We investigated the association between NO-related biomarkers and CVD outcomes in a healthy population, comparing adjustments using eGFR and measured GFR (mGFR).

**Methods:**

This 14-year longitudinal study evaluated 1575 healthy, middle-aged participants without pre-existing DM, CVD or kidney disease in the Renal Iohexol Clearance Survey (RENIS). Cox regression models assessed the effects of asymmetric dimethylarginine (ADMA), symmetric dimethylarginine (SDMA), arginine, citrulline and ornithine levels on CVD incidence and all-cause mortality. Analyses were adjusted for GFR using mGFR by iohexol clearance or eGFR based on serum creatinine (eGFR_c__rea_) or cystatin C (eGFR_c__ys_).

**Results:**

Elevated ADMA levels were associated with incident CVD across all GFR adjustment methods, with a hazard ratio (HR) of 1.21 [95% confidence interval (CI) 1.06–1.38] for mGFR. SDMA was associated with CVD when adjusting for eGFR_c__rea_ [HR 1.19 (95% CI 1.03–1.38)], not with mGFR or eGFR_c__ys_. Through all GFR methods, higher citrulline levels consistently correlated with CVD [HR 1.17 (95% CI 1.03–1.33) for mGFR]. No biomarkers were linked to all-cause mortality.

**Conclusions:**

In a healthy population, ADMA and citrulline were associated with incident CVD, regardless of the GFR adjustment method, while the association of SDMA depended on the method used.

## Graphical Abstract

**Graphical Abstract:**
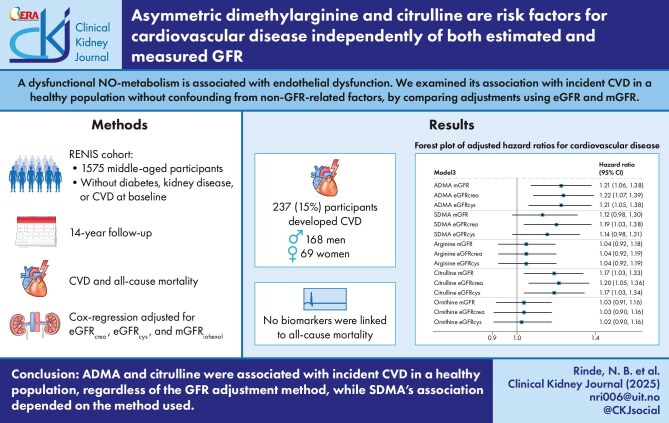

KEY LEARNING POINTS **What was known:**

- Nitric oxide (NO) metabolism is associated with cardiovascular disease (CVD) in populations with pre-existing risk factors for CVD and chronic kidney disease (CKD).
- Prior research has focused on populations with pre-existing or known risk for CVD, leaving the association in a healthy population unexplored.
- Previous studies adjusted for kidney function using estimates from creatinine, cystatin C or both, potentially allowing non-glomerular filtration rate (GFR)-related factors to influence the observed relationships.

**This study adds:**

- This study found that increased serum levels of asymmetric dimethylarginine (ADMA) and citrulline were associated with the development of CVD in a healthy population.
- The relationship of ADMA and citrulline with CVD was independent of the method used to assess kidney function.
- We validated previous findings of associations between SDMA and CVD when adjusting for eGFR based on creatinine. However, this association disappeared when adjusting for measured GFR or estimated GFR based on cystatin C.

**Potential impact:**

- In an aging population, ADMA and citrulline have the potential to identify individuals at high risk for CVD, enabling personalized prevention and management strategies.

## INTRODUCTION

Cardiovascular disease (CVD) continues to be a predominant cause of morbidity and mortality globally, accounting for a significant health burden [1, 2]. Traditional risk factors such as aging, diabetes mellitus, hypertension, hypercholesterolaemia, obesity and smoking are well-established contributors to the development of CVD [2, 3]. However, the role of non-traditional factors in CVD pathogenesis is not as well understood. Among these, nitric oxide (NO) may play a critical role. As a principal mediator of vasodilation, NO deficiency is closely linked to several mechanisms underlying cardiovascular pathology, including endothelial dysfunction, atherosclerosis, hypertension, oxidative stress, endothelial permeability and inflammation [4–6]. Research has explored NO precursors and NO inhibitors as potential independent risk factors for CVD and mortality in the general population [7–13].

Previous studies on these biomarkers have yielded partly conflicting results [8–20]. For instance, the Framingham Heart Study linked the NO inhibitor asymmetric dimethylarginine (ADMA) to all-cause mortality but not to CVD-related mortality [11], while other studies found ADMA predictive of cardiovascular and all-cause mortality [8, 12, 13]. The other inhibitor of NO, symmetric dimethylarginine (SDMA), has been associated with cardiovascular and all-cause mortality [9, 10, 17]. In contrast, arginine, a precursor of NO, was proposed as a possible protective agent against CVD [7], but it has previously shown no associations in other studies [8, 9, 11]. Increased citrulline levels, a precursor of arginine through the urea cycle with ornithine [21], have been associated with an increased risk of future CVD [22]. These studies from the general population included different proportions of participants with pre-existing CVD, diabetes and kidney disease in the cohorts, which may explain their different findings [8–20].

Individuals with a reduced glomerular filtration rate (GFR) share several pathophysiological mechanisms with NO deficiency and are often included in CVD studies. A common limitation in these studies is the adjustment of statistical analysis using GFR estimates based on creatinine [8–13], which is imprecise and may be influenced by non-GFR-related factors such as diet, muscle mass, inflammation, obesity and diabetes [23–25]. This complicates the investigation of the complex interactions between NO metabolism, kidney function and cardiovascular risk [26–28]. In particular, SDMA has been proposed as an alternative marker of GFR due to its strong correlation with measured GFR (mGFR) [29]. Thus, unbiased GFR adjustment is crucial in studies of NO-metabolism.

To our knowledge, no longitudinal studies have examined dimethylarginines and NO precursors as risk factors for mortality or CVD in a population-based cohort using adjustment for precise GFR measurements. This study aimed to investigate the associations between dimethylarginines and NO precursors with CVD in a population initially free of diabetes mellitus, kidney disease and CVD. Additionally, it sought to compare adjustments using estimated GFR (eGFR) with mGFR based on iohexol clearance.

## MATERIALS AND METHODS

### Study population

The Renal Iohexol Clearance Survey (RENIS) is a substudy of the Tromsø Study. This population-based prospective study conducts repeated health surveys of representative samples from Tromsø, in Northern Norway [30]. The baseline phase of the RENIS (RENIS-T6) was conducted between 2007 and 2009 and included 1627 individuals ages 50–62 years, as previously described [31]. Participants with self-reported kidney disease, CVD or diabetes were excluded.

In this present study, participants without baseline measurements of dimethylarginines and NO precursors (*n* = 3), with a haemoglobin A1c ≥48 mmol/mol (6.5%) (*n* = 29) or fasting glucose ≥7.0 mmol/l (*n* = 14) were excluded. Furthermore, during the medical records review by the RENIS Endpoint Committee, six participants who had initially self-reported as healthy were found to have a history of CVD, leading to their exclusion. Consequently, the final sample comprised 1575 participants.

All participants provided written informed consent. The Regional Ethics Committee of Northern Norway approved the study, which adhered to the Declaration of Helsinki.

### Baseline data and measurements

All participants completed a questionnaire about their current medication and smoking status. Body height and weight were measured to calculate body mass index (BMI), defined as weight in kilograms divided by the square of height in meters (kg/m^2^). As previously described, blood pressure (BP) was measured with an automated device [32]. Three first-void urine samples were collected on consecutive days before the other measurements. The albumin:creatinine ratio (ACR) was calculated for each urine specimen and the mean ACR value was used in the analyses [33]. All measurements were conducted in the morning at the Clinical Research Unit at the University Hospital of Northern Norway following an overnight fast. Fasting plasma samples were collected from a Teflon catheter inserted into an antecubital vein for biochemical analyses. Serum samples for glucose, creatinine, cystatin C, triglycerides and cholesterol were measured on the same day. Creatinine was measured using a standardized enzymatic assay [27]. Cystatin C was measured using a particle-enhanced turbidimetric immunoassay [27]. The Chronic Kidney Disease Epidemiology Collaboration equations from 2012 based on creatinine (eGFR_c__rea_) and cystatin C (eGFR_c__ys_) were used in the estimation of GFR [30].

### Dimethylarginines and NO precursors

The analysis of ADMA, SDMA, arginine, citrulline and ornithine within the RENIS cohort has been described in detail previously [27, 28] and in the supplementary materials. Quantification of the biomarkers in serum was performed with liquid chromatography tandem mass spectrometry (LC-MS/MS).

### Iohexol clearance

As previously described, GFR was measured in RENIS-T6 using single-sample plasma iohexol clearance [34], validated against gold-standard methods [31, 35]. For this procedure, a Teflon venous catheter was injected with 5 ml of iohexol (Omnipaque, 300 mg iodine/ml; Amersham Health, London, UK). The timing for measuring iohexol clearance post-injection was determined using Jacobsson's method [36]. The iohexol concentration was analysed using high-performance liquid chromatography [37]. The GFR was standardized to a body surface area of 1.73 m^2^. The body surface area was estimated using the equation of Du Bois and Du Bois [38].

### Outcomes

The RENIS Endpoint Committee conducted adjudication of the composite CVD outcome based on review of the medical records of the North Norwegian Regional Health Authority, and the follow-up period concluded on 1 January 2023. The composite CVD outcome included the first occurrence of myocardial infarction (MI), stroke, coronary revascularization procedure without concurrent infarction, diagnosis of stenosis in other arteries or sudden death without a non-CVD cause (described in detail in the supplementary material).

### Statistical analysis

The baseline data were dichotomized into groups based on CVD status, i.e. no CVD and incident CVD groups. Continuous variables were presented as mean ± standard deviation (SD) for normal data or median and interquartile range (IQR) for skewed variables. The differences between the CVD and non-CVD groups were tested with *t*-tests and chi-squared tests.

Cox proportional hazard regression models were employed to estimate the hazard ratio (HR) with a 95% confidence interval (CI) for the incidence of CVD associated with the biomarkers. Analyses included assessments for the main components of CVD: stroke, MI and other causes of CVD, in addition to a separate analysis of all-cause mortality associated with the biomarkers. The time from the initial entry in the RENIS was defined as the entry time, with the CVD event, death or censoring date as the exit time. The analyses comprised three models: Model 1 was adjusted for baseline age, sex and BMI; Model 2 included adjustments from Model 1 plus baseline office systolic BP, use of BP-lowering drugs [use of angiotensin-converting enzyme inhibitors, angiotensin receptor II blockers, diuretics, calcium blockers, beta-blockers or other antihypertensive medications (yes/no)], use of lipid-lowering drugs (yes/no), fasting glucose, total cholesterol, current smoking status (yes/no), ACR and C-reactive protein (CRP); and Model 3 incorporated all variables from Model 2 and further adjusted for mGFR. Analyses in Model 3 were also conducted using eGFR with creatinine or cystatin C instead of mGFR to compare different adjustment methods for kidney function. The HRs were visually presented through a forest plot. A directed acyclic graph (DAG) was employed for Model 3 to ensure comprehensive adjustment for confounding and to prevent bias from non-causal pathways; it is provided in Supplementary Fig. S2 [39].

Interaction terms between sex*biomarker, age*biomarker and BMI ≥30*biomarker was introduced for each of the biomarkers to explore potential interactions. A *P*-value <.05 was considered a statistically significant interaction and warranted sex-stratified Cox proportional hazards regression analyses for incident CVD for that biomarker.

Kaplan–Meier survival estimates assessed the relationship between NO precursors or dimethylarginine and CVD events over the study period, stratifying participants into tertiles by biomarker concentrations.

All statistical analysis was performed using Stata/MP version 18.0 (StataCorp, College Station, TX, USA).

## RESULTS

In our cohort of 1575 participants, 237 (15%) experienced a CVD event during a median follow-up of 14.1 years, whereas the first event occurred 5 months after the baseline measurements. Among these, 168 cases were men (22% of men) and 69 were women (8.6% of women). The baseline characteristics, dichotomized into CVD and non-CVD groups, are detailed in Table 1. Notably, the CVD group had significantly higher age, systolic and diastolic BP and levels of all biomarkers, triglyceride, high-density lipoprotein and low-density lipoprotein (*P* < .05). Further, the group with CVD outcomes had a higher proportion of current smokers and individuals on antihypertensive and lipid-lowering medication (*P* < .05). Table 2 presents descriptive statistics of the biomarkers ADMA, SDMA, arginine, citrulline and ornithine with mean, median, SD and minimum and maximum values.

**Table 1:** Baseline characteristics with the RENIS cohort dichotomized into those who experienced a CVD outcome during follow-up and those who did not.

Characteristics	All patients (*N* = 1575)	No CVD (*n* = 1338)	CVD (*n* = 237)	*P*-value
Age (years)	58.0 (3.8)	57.9 (3.9)	58.8 (3.6)	<0.001
Male, *n* (%)	772 (49)	604 (45)	168 (71)	<0.001
BMI (kg/m^2^)	27.2 (4.0)	27.2 (4.0)	27.5 (3.8)	0.12
mGFR (ml/min/1.73 m^2^)	93.8 (14.2)	93.8 (14.1)	94.0 (14.8)	0.40
eGFR_c__rea_ (ml/min/1.73 m^2^)	94.8 (9.6)	94.9 (9.6)	94.3 (9.5)	0.20
eGFR_c__ys_ (ml/min/1.73 m^2^)	105.4 (12.3)	105.6 (12.1)	104.4 (13.8)	0.08
Systolic BP (mmHg)	129.3 (17.6)	128.2 (17.2)	135.7 (18.4)	<0.001
Diastolic BP (mmHg)	83.3 (9.8)	82.9 (9.7)	86.1 (9.8)	<0.001
Total cholesterol (mmol/l)	5.6 (0.9)	5.6 (0.9)	5.7 (1.0)	0.32
HDL cholesterol (mmol/l)	1.5 (0.4)	1.6 (0.4)	1.4 (0.4)	<0.001
LDL cholesterol (mmol/l)	3.7 (0.9)	3.6 (0.8)	3.8 (0.9)	0.01
Fasting triglycerides (mmol/l)	1.2 (0.7)	1.2 (0.6)	1.3 (0.8)	<0.001
Fasting glucose (mmol/l)	5.3 (0.5)	5.3 (0.5)	5.4 (0.5)	0.07
ADMA (µmol/l)	0.427 (0.1)	0.426 (0.1)	0.435 (0.1)	0.007
SDMA (µmol/l)	0.62 (0.1)	0.616 (0.1)	0.637 (0.1)	0.003
Arginine (µmol/l)	93.6 (16.9)	93.2 (16.5)	95.9 (18.9)	0.01
Citrulline (µmol/l)	21.3 (6.2)	21.1 (6.1)	22.4 (6.4)	0.001
Ornithine (µmol/l)	62.4 (14.1)	62.1 (13.9)	64.3 (15.2)	0.01
CRP (mg/l), median (IQR)	1.2 (0.6–2.2)	1.2 (0.6–2.2)	1.3 (0.7–2.1)	0.39
ACR (mg/mmol), median (IQR)	0.2 (0.1–0.5)	0.2 (0.1–0.5)	0.3 (0.1–0.6)	0.10
Antihypertensive medication, *n* (%)	280 (18)	224 (17)	56 (24)	0.01
Lipid-lowering medication, *n* (%)	97 (6)	74 (6)	23 (10)	0.01
Current smoker, *n* (%)	333 (21)	257 (19)	76 (32)	<0.001

Data are presented as mean (SD) unless stated otherwise.

**Table 2:** Descriptive statistics of the biomarkers with mean, median, SD, minimum and maximum values.

Biomarkers	Mean	Median	SD	Minimum	Maximum
ADMA	0.43	0.43	0.06	0.13	0.68
SDMA	0.62	0.62	0.10	0.23	1.05
Arginine	93.63	92.37	16.89	28.04	153.47
Citrulline	21.31	20.80	6.17	0.94	49.60
Ornithine	62.43	60.78	14.15	22.61	152.88

**Figure 1:**
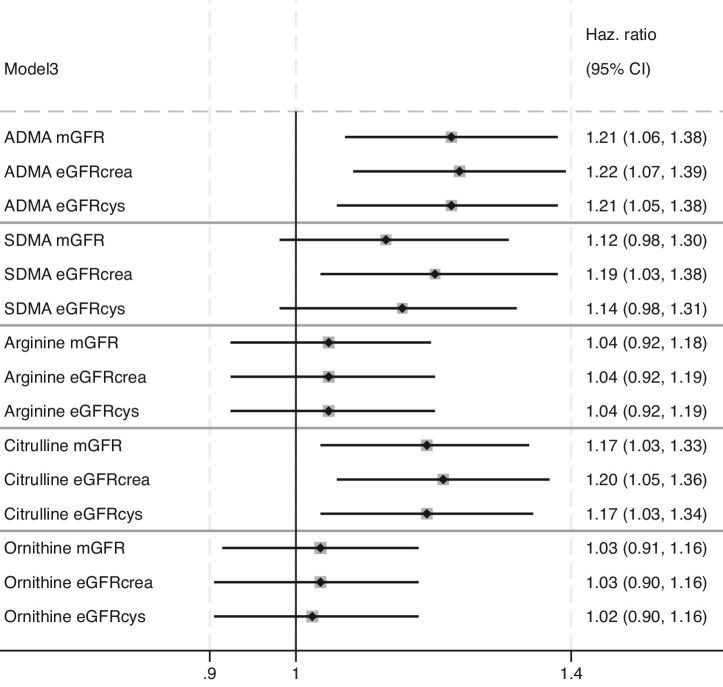
Forest plot of adjusted HRs for CVD events. Cox proportional hazards regression models were employed to estimate the HR per SD increase with a 95% CI adjusted for Model 3 with different GFRs (mGFR, eGFR_crea_ or eGFR_cys_). Model 3: Adjustments for baseline age; sex; BMI; systolic BP; use of angiotensin-converting enzyme inhibitors, angiotensin receptor II blockers, diuretics, calcium blockers, beta-blockers or other antihypertensive medications (yes/no); use of lipid-lowering drugs (yes/no); fasting glucose; cholesterol; smoking status (yes/no); CRP; ACR and kidney function with GFR (mGFR, eGFR_crea_ or eGFR_cys_).

Table 3 presents the Cox regression analyses of dimethylarginines and NO precursors and their association with the CVD outcome. Fig. 1 shows HRs for Model 3 comparing different methods of kidney function adjustment. Per a 1 SD higher baseline ADMA, there was a 21% increased HR for incident CVD [HR 1.21 (95% CI 1.06–1.38)] in Model 3, adjusted for mGFR. Similarly, each SD with higher baseline citrulline levels was associated with a 17% higher HR for incident CVD [HR 1.17 (95% CI 1.03–1.33)] in Model 3 adjusted for mGFR. Similar results for ADMA and citrulline were found when eGFR was included in the models instead of mGFR (Fig. 1). Higher baseline SDMA levels were associated with CVD incidence in Model 3 only when eGFR_crea_ was the method of kidney function adjustment [HR 1.19 per 1 SD increase in SDMA (95% CI 1.03–1.38), *P* = .02], not with mGFR or eGFR_c__ys_ [HR 1.12 (95% CI 0.98–1.30), *P* = .11 and HR 1.13 (95% CI 0.98–1.31), *P* = .08, respectively, in Fig. 1]. We found no association between incident CVD and either arginine or ornithine, regardless of the method of kidney function adjustment.

**Table 3:** Cox regression for events of CVD adjusted for mGFR in Model 3.

Biomarkers	Model 1, HR per SD (95% CI)	*P*-value	Model 2, HR per SD (95% CI)	*P*-value	Model 3, HR per SD (95% CI)	*P*-value
ADMA	1.16 (1.03–1.32)	.019	1.22 (1.07–1.40)	.003	1.21 (1.06–1.38)	.006
SDMA	1.10 (0.97–1.24)	.16	1.16 (1.02–1.31)	.02	1.12 (0.98–1.30)	.11
Arginine	1.11 (0.98–1.25)	.12	1.05 (0.93–1.19)	.45	1.04 (0.92–1.18)	.50
Citrulline	1.19 (1.05–1.35)	.007	1.19 (1.05–1.35)	.007	1.17 (1.03–1.33)	.02
Ornithine	1.09 (0.96–1.23)	.19	1.03 (0.91–1.16)	.67	1.03 (0.91–1.16)	.69

Model 1: adjusted for age, sex and BMI. Model 2: adjusted for model 1 and systolic BP; use of angiotensin-converting enzyme inhibitors, angiotensin receptor II blockers, diuretics, calcium blockers, beta-blockers or other antihypertensive medications (yes/no); fasting glucose; cholesterol; smoking status (yes/no); lipid-lowering drug (yes/no); CRP and ACR. Model 3: adjusted for model 2 and mGFR. Each row represents a separate regression model.

Supplementary Table S2 shows the association between all-cause mortality and the biomarkers; no associations were observed with any of the biomarkers.

**Figure 2:**
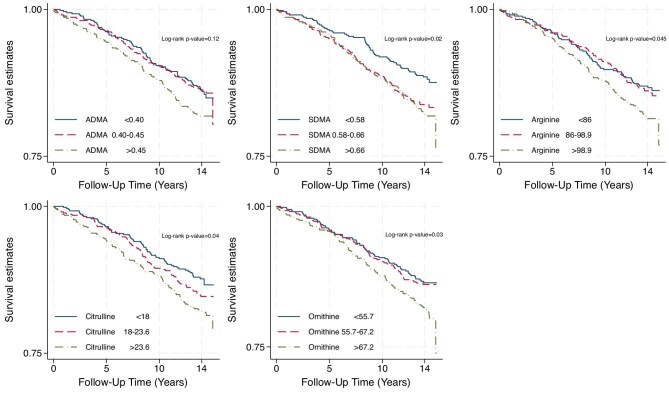
Kaplan-Meier survival analyses for cardiovascular disease for each of the ADMA (a), SDMA (b), arginine (c), citrulline (d), and ornithine (e) divided into tertiles based on their concentrations (n=525)

The 14-year unadjusted survival rate for incident CVD, stratified by tertiles of biomarker concentrations, is shown in Fig. 2 using a Kaplan–Meier curve. The curve reveals a lower event-free rate among participants in the highest tertile of the biomarker concentrations. The differences in survival rates across tertiles for SDMA, arginine, citrulline and ornithine were statistically significant, with logrank *P*-values <.05.

An interaction between ornithine and sex in Model 3 was identified for all kidney function assessments (*P* = .01), with sex-stratified Cox regression analyses presented in Supplementary Table S1. While no statistically significant association was found between ornithine levels and incident CVD in men, an increased risk of 28% of incident CVD per SD increase in ornithine was observed in women when adjusting GFR [mGFR HR 1.28 (95% CI 1.03–1.60), eGFR_c__rea_ HR 1.29 (95% CI 1.04–1.61) and eGFR_c__ys_ HR 1.28 (95% CI 1.02–1.60)]. No interaction was observed between the other biomarkers and sex, age or BMI ≥30.

## DISCUSSION

In a representative sample of a European population without diabetes, kidney disease and CVD, we observed that higher baseline levels of ADMA and citrulline were associated with an elevated risk of CVD and that the association was independent of the method of GFR assessment. The association between SDMA and CVD was only statistically significant when adjusted for eGFR_crea_, not when adjusted for mGFR or eGFR_cys_. We found no association between arginine or ornithine levels and CVD.

Our finding aligns with previous studies linking ADMA levels to CVD in the general population and among individuals with kidney disease [8, 11, 18, 40]. This suggests that elevated ADMA levels may serve as a risk factor for CVD independent of previous CVD, kidney disease and diabetes. Previous studies have shown that higher ADMA reduces NO bioavailability and further affects endothelial dysfunction, which may explain the pathophysiological link between ADMA and CVD [4, 13, 41]. Furthermore, since the associations remained almost identical regardless of adjustment for mGFR or eGFR, previous findings using eGFR were probably not biased by non-GFR factors [24, 25].

Although less emphasized in CVD research, citrulline, a precursor of arginine, has been hypothesized to enhance the availability of NO. Oral citrulline supplementation has been explored for its potential to be more effective than arginine supplementation in improving NO-mediated vascular function; however, the results have varied [42, 43]. Definitive conclusions regarding the therapeutic efficacy of oral supplements remain elusive due to the small sample sizes of the existing clinical studies on the topic [43]. A recent observational study investigated the long-term mortality in hospitalized CVD patients and found no correlation with citrulline levels [44]. This finding differs from our results, which identified a relationship between citrulline levels and CVD risk. Two longitudinal studies—one conducted in a population with pre-existing CVD and the other without—found associations between elevated citrulline and increased CVD risk [22, 45]. The exact reason for the observed risk remains unclear in the literature. However, it has been suggested that elevated citrulline may reflect increased ADMA levels, as citrulline is a byproduct of ADMA degradation [5], or may indicate an upregulation of citrulline production as a compensatory mechanism to enhance NO bioavailability in response to an NO shortage [42].

The role of SDMA also warrants attention, as previous studies have associated an increased risk of CVD and all-cause mortality in cohorts from the general population that adjusted for creatinine-based eGFR [9, 10, 17, 18, 46]. The biological functions of SDMA are not yet fully understood, but it is recognized that SDMA's connection to CVD may operate through several mechanisms, including the modulation of high-density lipoprotein, the production of reactive oxygen species and the activation of pro-inflammatory pathways [46, 47]. However, in our analysis, the association between SDMA and CVD was no longer statistically significant when adjusting for mGFR or eGFR_c__ys_ instead of eGFR_c__rea_. Several reasons may explain this result: first, non-GFR-related factors influencing eGFR may affect the association between SDMA and CVD [27, 28]; second, the result may represent a true null finding, suggesting eGFR_c__rea_ is a unreliable proxy for the actual GFR when investigating SDMA's effect on CVD; and third, the observation may be a type I error, i.e. a false positive association [48]. This finding suggests that future studies exploring the association between SDMA and CVD should use mGFR or eGFR_c__ys_ when adjusting for kidney function, not eGFR_c__rea_.

Arginine is thought to enhance the bioavailability of NO, potentially exerting a protective effect against CVD [7]. However, consistent with previous research [8, 9, 11], our results did not show an association between arginine levels and the incidence of CVD.

No association with CVD incidence was observed in the overall population regarding ornithine. However, there was a statistically significant interaction between ornithine and sex, and stratified analysis revealed sex-specific differences, with higher baseline levels of ornithine associated with incident CVD in females but not in males. This discrepancy may indicate an underlying biological difference that warrants further investigation.

The strengths of this study lie in the accuracy of kidney function assessment, the method of analysing the biomarkers, the prospective study design and the long follow-up time. Using mGFR with iohexol clearance minimizes the potential confounding effects of non-GFR-related factors [27], enhancing the reliability of the findings.

The generalizability of this study is limited because only healthy, middle-aged North European participants were enrolled. Further, the healthy nature of the cohort resulted in fewer observed events of CVD and all-cause mortality. This is not unusual for study populations, as they tend to be healthier than those not participating [49]. The observational design of the study does not permit inferences about causality.

## CONCLUSION

The results of this study show an association between NO metabolism and CVD in a healthy population, even after adjusting for mGFR. Higher levels of ADMA and citrulline were associated with an increased risk of CVD. Future studies exploring the relationship between dimethylarginines and CVD can generally use eGFR for kidney function adjustment without introducing significant bias, except potentially in the case of SDMA.

## Supplementary Material

sfaf181_Supplemental_File

## Data Availability

The data underlying this article cannot be shared publicly because this was not included in the research permission due to ethical considerations and the privacy of individuals who participated in the study. The data can be shared on request as part of a research collaboration. Please contact the corresponding author.
