# Supplementary material for: Asymmetric dimethylarginine and citrulline are risk factors for cardiovascular disease independently of both estimated and measured GFR
Source: Clin Kidney J. 2025 Jun 13;18(7):sfaf181. doi: 10.1093/ckj/sfaf181 (PMC12241856; doi:10.1093/ckj/sfaf181)
Supplement: sfaf181_Supplemental_File [file sfaf181_supplemental_file.docx]

Supplementary Material

# Asymmetric dimethylarginine (ADMA) and citrulline are risk factors for cardiovascular disease independently of both estimated and measured GFR

Nikoline B. Rinde^1,2^, Toralf Melsom^1,2^, Ole Martin Fuskevåg^3,4^, Bjørn O. Eriksen^1,2^ and Jon Viljar Norvik^1,2^

1 Metabolic and Renal Research Group, UiT The Arctic University of Norway, Tromsø, Norway.

2 Section of Nephrology, University Hospital of North Norway, Tromsø, Norway.

3 Division of Diagnostic Services, Department of Laboratory Medicine, University Hospital of North Norway, Tromsø, Norway.

4 Department of Clinical Medicine, UiT The Arctic University of Norway, Tromsø, Norway

Running Head: NO-metabolism, CVD, and kidney function

Contents

[The composite cardiovascular outcomes 3](#_Toc196298108)

[Material analyses of dimethylarginines and nitric oxide precursors 4](#_Toc196298109)

[Table S1: Cox regression for all-cause mortality 4](#_Toc196298110)

[Table S2: Cox regression for CVD with sex differences 6](#_Toc196298111)

[Table S3: Missing study variables in the Renal Iohexol Clearance Survey 7](#_Toc196298112)

[Table S4: Cardiovascular outcomes in the RENIS cohort 7](#_Toc196298113)

[Table S5: Competing risk with Cox regression using Fine-Gray subhazard model for events of cardiovascular disease adjusted for mGFR in model 3. 8](#_Toc196298114)

[Table S6: Cox regression with composite outcomes for CVD: stroke, myocardial infarction, and other causes of CVD. 9](#_Toc196298115)

[Table S7. Spearman correlation coefficients between ADMA, SDMA, and citrulline and the kidney function 10](#_Toc196298116)

[Figure S1: Directed acyclic graph (DAG) 11](#_Toc196298117)

[Figure S2. Study population selection 12](#_Toc196298118)

[Figure S3: Histogram of study participants distributed on biomarkers concentration: 13](#_Toc196298119)

## The composite cardiovascular outcomes

The RENIS Endpoint Committee conducted adjudication of the composite CVD outcome based on the review of the medical records of the North Norwegian Regional Health Authority, and the follow-up period concluded on January 1st, 2023. The follow-up duration was calculated from the participants’ initial visit to RENIS until the date of an event or the censoring date. The observations were censored at the time of death from a non-CVD cause or at the end of the study. In instances where a participant experienced multiple cardiovascular events, the first event was recorded as the primary CVD event.

The composite CVD outcome was defined as the first occurrence of either fatal or nonfatal myocardial infarction or stroke, coronary revascularization procedure without concurrent infarction, the diagnosis of stenosis in other arteries (carotid, renal, peripheral, or other) or sudden unexpected death without identifiable non-CVD cause.

Myocardial infarction (MI) was classified according to the World Health Organization MONICA (Monitoring of Trends and Determinants in Cardiovascular Disease)/MORGAM (MONICA Risk, Genetics, Archiving and Monograph Project) criteria (1). This classification involves a combination of clinical symptoms and signs, electrocardiogram findings, and elevated levels of specific cardiac biomarkers such as ASAT, CK-MB, and troponin. In this study, only those events that were definitely or possibly acute MI (coded 1 or 2) under the MONICA criteria were included as MI outcomes.

Stroke was defined to include ischemic stroke, subarachnoid hemorrhage, intraparenchymal hemorrhage, other types of brain hemorrhage, or strokes of unknown type. Conditions such as subdural and epidural hematomas were not classified as strokes. The diagnosis of stroke was based on clinical symptoms and signs and confirmed by computerized tomography or magnetic resonance imaging.

Data on all-cause mortality were obtained from the Norwegian Cause of Death Registry. For individuals who died in a hospital within the North Norwegian Health Region, the cause of death was determined from their medical records. For deaths outside this region, the cause of death taken from the Norwegian Cause of Death Registry was utilized. Deaths with no information about the cause in either medical records or the Death Registry were classified as non-CVD deaths.

## Material analyses of dimethylarginines and nitric oxide precursors

Quantification of the biomarkers ADMA, SDMA, arginine, citrulline, and ornithine in serum was performed with liquid chromatography tandem mass spectrometry (LC-MS/MS). The analytical system comprised of a Waters Acquity UPLC *I*-Class FTN system with an autosampler and a binary solvent delivery system (Waters, Milford, MA) interfaced to a Waters Xevo TQ-X benchtop tandem quadrupole mass spectrometer (Waters, Manchester, UK). The reference material for ADMA, SDMA, arginine, citrulline, and ornithine were sourced from Sigma-Aldrich (St. Louis, Mo., USA), while their labeled isotopes were acquired from Toronto Research Chemicals (Ontario, Canada). The analytical precision was validated, demonstrating an interday coefficient of variation of less than 8% across three separate days for each analyte. Serum samples used to measure ADMA, SDMA, arginine, citrulline, and ornithine levels were stored at -80˚C and thawed on the analysis day.

Table S1: Cox regression for all-cause mortality adjusted for mGFR and eGFR with creatinine and cystatin C base equations. (n=120)

|  | Model 1 |  | Model 2 |  | Model 3 |  |
| --- | --- | --- | --- | --- | --- | --- |
|  | HR per SD (95% CI) | P-value | HR per SD (95% CI) | P-value | HR per SD (95% CI) | P-value |
| ADMA |  |  |  |  |  |  |
| mGFR | 1.02 (0.85-1.22) | 0.838 | 0.98 (0.81-1.17) | 0.803 | 0.99 (0.82-1.19) | 0.894 |
| eGFR_crea_ |  |  |  |  | 1.00 (0.83-1.20) | 0.998 |
| eGFR_cys_ |  |  |  |  | 1.00 (0.83-1.21) | 0.960 |
| SDMA |  |  |  |  |  |  |
| mGFR | 0.95 (0.79-1.14) | 0.569 | 0.93 (0.78-1.12) | 0.456 | 0.95 (0.77-1.17) | 0.642 |
| eGFR_crea_ |  |  |  |  | 1.08 (0.88-1.34) | 0.445 |
| eGFR_cys_ |  |  |  |  | 0.98 (0.80-1.20) | 0.833 |
| Arginine |  |  |  |  |  |  |
| mGFR | 0.92 (0.77-1.10) | 0.387 | 0.88 (0.73-1.05) | 0.146 | 0.88 (0.73-1.05) | 0.147 |
| eGFR_crea_ |  |  |  |  | 0.89 (0.75-1.06) | 0.199 |
| eGFR_cys_ |  |  |  |  | 0.88 (0.73-1.05) | 0.146 |
| Citrulline |  |  |  |  |  |  |
| mGFR | 0.92 (0.76-1.11) | 0.404 | 0.90 (0.75-1.09) | 0.300 | 0.91 (0.75-1.11) | 0.361 |
| eGFR_crea_ |  |  |  |  | 0.97 (0.79-1.17) | 0.724 |
| eGFR_cys_ |  |  |  |  | 0.92 (0.76-1.12) | 0.415 |
| Ornithine |  |  |  |  |  |  |
| mGFR | 1.04 (0.87-1.24) | 0.670 | 0.98 (0.82-1.17) | 0.805 | 0.98 (0.82-1.17) | 0.821 |
| eGFR_crea_ |  |  |  |  | 0.98 (0.82-1.17) | 0.846 |
| eGFR_cys_ |  |  |  |  | 0.98 (0.82-1.18) | 0.861 |

ADMA, asymmetric dimethylarginine; CI, confidence interval; eGFR_Crea_, estimated glomerular filtration rate based on creatinine; eGFR_Cys_, estimated glomerular filtration rate based on cystatin C; HR, hazard ratio; mGFR, measured glomerular filtration rate; SDMA, symmetric dimethylarginine.
Model 1: adjusted for age, sex, and BMI. Model 2: model 1 and adjusted for systolic blood pressure, use of angiotensin-converting enzyme inhibitors, angiotensin receptor II blockers, diuretics, calcium blockers, beta-blockers, or other antihypertensive medications (yes/no), fasting glucose, cholesterol, smoking status (yes/no), lipid-lowering drug (yes/no), C-reactive protein, and albumin-to-creatinine ratio. Model 3: model 2 and adjusted for GFR either with measured iohexol or estimated with creatinine or cystatin C.
Each row represents a separate regression model.

Table S2: Cox regression for CVD with sex differences for ornithine.

|  | Model 1 |  | Model 2 |  | Model 3 |  |
| --- | --- | --- | --- | --- | --- | --- |
|  | HR per SD (95% CI) | P-value | HR per SD (95% CI) | P-value | HR per SD (95% CI) | P-value |
| All |  |  |  |  |  |  |
| mGFR | 1.09 (0.96-1.23) | 0.193 | 1.03 (0.91-1.16) | 0.671 | 1.03 (0.91-1.16) | 0.691 |
| eGFR_crea_ |  |  |  |  | 1.03 (0.91-1.16) | 0.674 |
| eGFR_cys_ |  |  |  |  | 1.02 (0.91-1.16) | 0.696 |
| Male |  |  |  |  |  |  |
| mGFR | 0.96 (0.82-1.12) | 0.623 | 0.93 (0.80-1.08) | 0.367 | 0.93 (0.80-1.09) | 0.373 |
| eGFR_crea_ |  |  |  |  | 0.93 (0.80-1.08) | 0.367 |
| eGFR_cys_ |  |  |  |  | 0.93 (0.80-1.08) | 0.367 |
| Female |  |  |  |  |  |  |
| mGFR | 1.40 (1.14-1.73) | 0.002 | 1.30 (1.04-1.62) | 0.021 | 1.28 (1.03-1.60) | 0.028 |
| eGFR_crea_ |  |  |  |  | 1.29 (1.04-1.61) | 0.023 |
| eGFR_cys_ |  |  |  |  | 1.28 (1.02-1.60) | 0.032 |

CI, confidence interval; eGFR_Crea_, estimated glomerular filtration rate based on creatinine; eGFR_Cys_, estimated glomerular filtration rate based on cystatin C; HR, hazard ratio; mGFR, measured glomerular filtration rate; SDMA, symmetric dimethylarginine.
Model 1: adjusted for age, sex, and BMI. Model 2: model 1 and adjusted for systolic blood pressure, use of angiotensin-converting enzyme inhibitors, angiotensin receptor II blockers, diuretics, calcium blockers, beta-blockers, or other antihypertensive medications (yes/no), fasting glucose, cholesterol, smoking status (yes/no), lipid-lowering drug (yes/no), C-reactive protein, and albumin-to-creatinine ratio. Model 3: model 2 and adjusted for GFR either with measured iohexol or estimated with creatinine or cystatin C.
Each row represents a separate regression model.

## Table S3: Missing study variables in the Renal Iohexol Clearance Survey

| Variable |  | Number (%) |
| --- | --- | --- |
| Urinary albumin-creatinine ratio |  | 5 (0.3 %) |
| Smoking |  | 3 (0.2 %) |
| C-reactive protein |  | 17 (1 %) |
| Complete cases |  | 1550 (98.4 %) |
| Total |  | 1575 (100 %) |

## Table S4: Cardiovascular outcomes in the RENIS cohort

| All cardiovascular outcomes | | |  | 237 (15 %) |  |  |
| --- | --- | --- | --- | --- | --- | --- |
|  | Coronary heart disease | |  |  | 131 (8.3 %) |  |
|  |  | Acute myocardial infarction | | |  | 53 (33.7 %) |
|  |  | Percutaneous coronary intervention^a^ | | |  | 54 (34.3 %) |
|  |  | Aortacoronary bypass surgery^a^ | | |  | 21 (1.3 %) |
|  |  | Sudden death | |  |  | 3 (0.2 %) |
|  | Stroke |  |  |  | 63 (4 %) |  |
|  | Other cardiovascular disease | | |  | 43 (2.7 %) |  |
| Total number of participants | | |  | 1575 (100 %) |  |  |

RENIS, the Renal Iohexol Clearance Survey.
^a^Coronary revascularization procedure without concurrent myocardial infarction.

## Table S5: Competing risk with Cox regression using Fine-Gray subhazard model for events of cardiovascular disease adjusted for mGFR in model 3.

|  | Model 1 |  | Model 2 |  | Model 3 |  |
| --- | --- | --- | --- | --- | --- | --- |
|  | SHR per SD (95% CI) | P-value | SHR per SD (95% CI) | P-value | SHR per SD (95% CI) | P-value |
| ADMA | 1.16 (1.02-1.33) | 0.02 | 1.21 (1.06-1.39) | 0.006 | 1.19 (1.04-1.37) | 0.012 |
| SDMA | 1.10 (0.97-1.25) | 0.16 | 1.16 (1.03-1.31) | 0.02 | 1.13 (0.99-1.30) | 0.07 |
| Arginine | 1.11 (0.97-1.27) | 0.14 | 1.05 (0.93-1.19) | 0.45 | 1.04 (0.92-1.18) | 0.50 |
| Citrulline | 1.20 (1.05-1.36) | 0.007 | 1.20 (1.05-1.36) | 0.007 | 1.18 (1.03-1.34) | 0.02 |
| Ornithine | 1.09 (0.96-1.23) | 0.18 | 1.03 (0.92-1.16) | 0.59 | 1.03 (0.92-1.16) | 0.61 |

ADMA, asymmetric dimethylarginine; CI, confidence interval; SHR, subhazard ratio; mGFR, measured glomerular filtration rate; SDMA, symmetric dimethylarginine.
Model 1: adjusted for age, sex, and BMI. Model 2: adjusted for model 1 and systolic blood pressure, use of angiotensin-converting enzyme inhibitors, angiotensin receptor II blockers, diuretics, calcium blockers, beta-blockers, or other antihypertensive medications (yes/no), fasting glucose, cholesterol, smoking status (yes/no), lipid-lowering drug (yes/no), C-reactive protein, and albumin-to-creatinine ratio. Model 3: adjusted for model 2 and mGFR.
Each row represents a separate regression model.

## Table S6: Cox regression with composite outcomes for CVD: stroke, myocardial infarction, and other causes of CVD.

|  | Stroke |  | Infarction |  | Other CVD |  |
| --- | --- | --- | --- | --- | --- | --- |
|  | HR (95% CI) | P-value | HR (95% CI) | P-value | HR (95% CI) | P-value |
| ADMA |  |  |  |  |  |  |
| mGFR | 1.10 (0.87-1.39) | 0.43 | 1.29 (1.00-1.68) | 0.05 | 1.15 (0.95-1.40) | 0.16 |
| eGFR_crea_ | 1.13 (0.89-1.42) | 0.31 | 1.30 (1.01-1.69) | 0.044 | 1.16 (0.96-1.41) | 0.13 |
| eGFR_cys_ | 1.11 (0.88-1.42) | 0.38 | 1.29 (0.99-1.68) | 0.06 | 1.14 (0.94-1.40) | 0.19 |
| SDMA |  |  |  |  |  |  |
| mGFR | 1.15 (0.89-1.49) | 0.27 | 1.22 (0.91-1.64) | 0.19 | 1.02 (0.83-1.27) | 0.84 |
| eGFR_crea_ | 1.31 (1.01-1.70) | 0.046 | 1.17 (0.87-1.57) | 0.29 | 1.07 (0.86-1.33) | 0.54 |
| eGFR_cys_ | 1.21 (0.94-1.57) | 0.14 | 1.22 (0.92-1.64) | 0.17 | 0.99 (0.80-1.22) | 0.90 |
| Arginine |  |  |  |  |  |  |
| mGFR | 0.88 (0.70-1.10) | 0.26 | 1.02 (0.79-1.32) | 0.88 | 1.19 (0.99-1.42) | 0.06 |
| eGFR_crea_ | 0.88 (0.70-1.10) | 0.27 | 1.01 (0.78-1.30) | 0.95 | 1.18 (0.98-1.41) | 0.08 |
| eGFR_cys_ | 0.88 (0.70-1.10) | 0.26 | 1.02 (0.79-1.32) | 0.87 | 1.17 (0.97-1.41) | 0.09 |
| Citrulline |  |  |  |  |  |  |
| mGFR | 1.17 (0.93-1.47) | 0.19 | 1.19 (0.92-1.53) | 0.20 | 1.09 (0.90-1.32) | 0.39 |
| eGFR_crea_ | 1.22 (0.97-1.54) | 0.10 | 1.15 (0.89-1.50) | 0.29 | 1.11 (0.91-1.35) | 0.30 |
| eGFR_cys_ | 1.19 (0.94-1.50) | 0.15 | 1.19 (0.91-1.54) | 0.20 | 1.07 (0.88-1.30) | 0.48 |
| Ornithine |  |  |  |  |  |  |
| mGFR | 0.91 (0.72-1.15) | 0.44 | 1.20 (0.95-1.52) | 0.13 | 1.01 (0.85-1.21) | 0.90 |
| eGFR_crea_ | 0.92 (0.73-1.16) | 0.48 | 1.20 (0.94-1.51) | 0.14 | 1.00 (0.84-1.20) | 1.00 |
| eGFR_cys_ | 0.92 (0.72-1.16) | 0.46 | 1.20 (0.95-1.53) | 0.12 | 1.00 (0.83-1.20) | 1.00 |

ADMA, asymmetric dimethylarginine; CI, confidence interval; HR, hazard ratio; mGFR, measured glomerular filtration rate; SDMA, symmetric dimethylarginine.
Model 3: adjusted for age, sex, BMI, systolic blood pressure, use of angiotensin-converting enzyme inhibitors, angiotensin receptor II blockers, diuretics, calcium blockers, beta-blockers, or other antihypertensive medications (yes/no), fasting glucose, cholesterol, smoking status (yes/no), lipid-lowering drug (yes/no), C-reactive protein, and albumin-to-creatinine ratio.

Each row represents a separate regression analysis with different kidney function assessment method.

## Table S7. Spearman correlation coefficients between ADMA, SDMA, and citrulline and the kidney function

|  | ADMA | | SDMA | | Arginine | | Citrulline | | Ornithine | |
| --- | --- | --- | --- | --- | --- | --- | --- | --- | --- | --- |
|  | Rho | P-value | Rho | P-value | Rho | P-value | Rho | P-value | Rho | P-value |
| ADMA |  |  |  |  |  |  |  |  |  |  |
| SDMA | 0.47 | <0.001 |  |  |  |  |  |  |  |  |
| Arginine | 0.34 | <0.001 | 0.14 | <0.001 |  |  |  |  |  |  |
| Citrulline | 0.37 | <0.001 | 0.36 | <0.001 | 0.19 | <0.001 |  |  |  |  |
| Ornithine | 0.37 | <0.001 | 0.18 | <0.001 | 0.18 | <0.001 | 0.28 | <0.001 |  |  |
| mGFR | -0.19 | <0.001 | -0.44 | <0.001 | 0.05 | 0.06 | -0.20 | <0.001 | -0.03 | 0.24 |
| eGFRcrea | -0.11 | <0.001 | -0.49 | <0.001 | -0.03 | 0.22 | -0.24 | <0.001 | -0.03 | 0.32 |
| eGFRcys | -0.29 | <0.001 | -0.38 | <0.001 | -0.03 | 0.25 | -0.19 | <0.001 | -0.08 | 0.003 |

Spearman’s test was used to estimate the pairwise correlation between ADMA, SDMA, citrulline, and GFR assessment methods.

Figure S1: Directed acyclic graph (DAG) Model 3


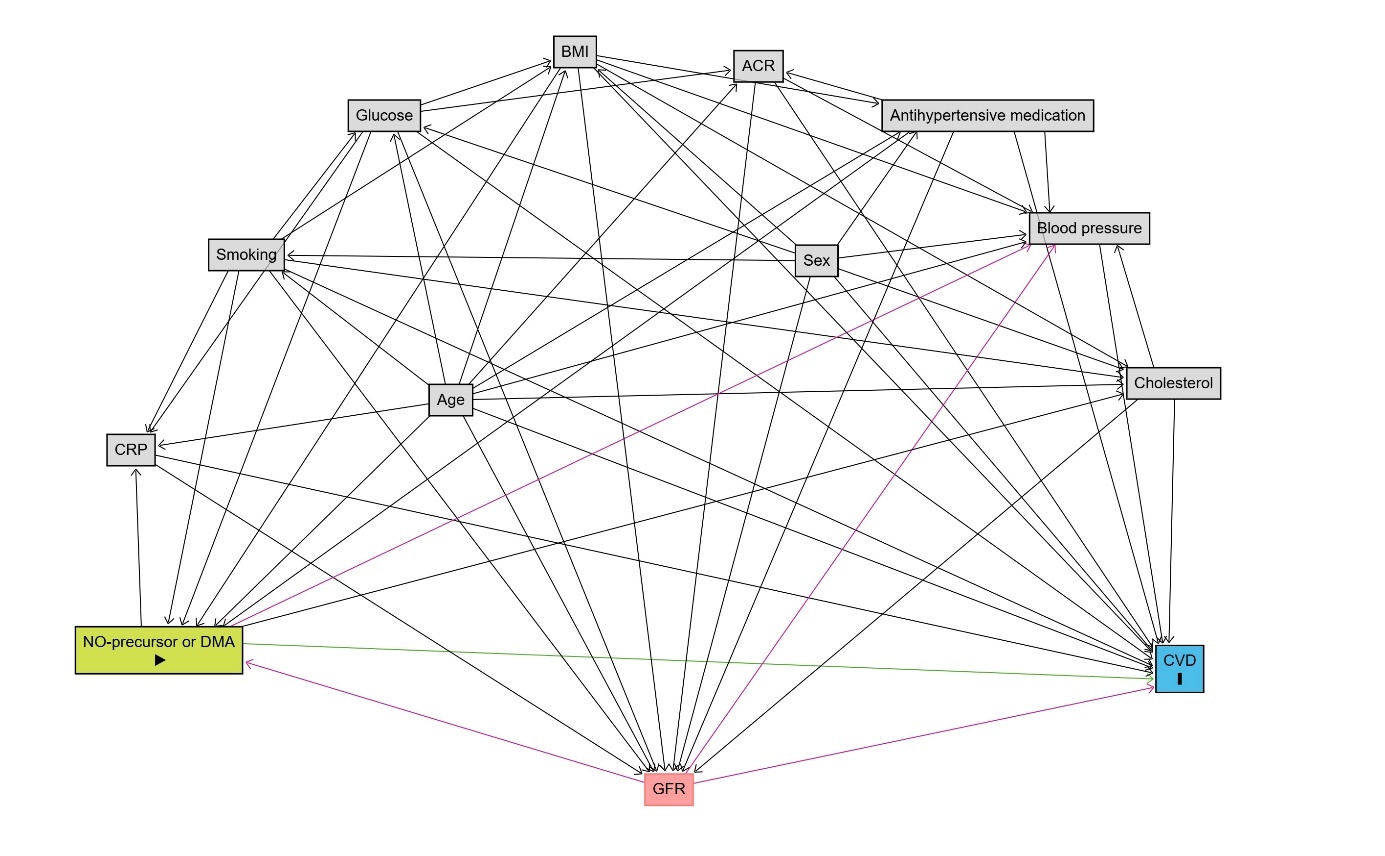


DAGs (Directed Acyclic Graphs) provide a visual representation of the assumptions of the structure and relationships between independent variables (ADMA, SDMA, arginine, citrulline, and ornithine) and the dependent variable (CVD). This visual representation helps clarify theoretical pathways, emphasizing potential confounders and mediators, thereby providing a clearer understanding of the underlying assumptions in our analysis. In the DAGs, Exposure was denoted by a green box, outcome by a blue box, and variables adjusted for are in grey boxes. Causal pathways were marked with green lines. The red box visualizes the ancestor of exposure and outcome. It marks the need for adjustment to find exposure's direct effect on outcome. In Model 3, we ended up adjusting age, sex, BMI, baseline ambulatory blood systolic pressure, the use of antihypertensive medication, fasting glucose, cholesterol, current smoking, ACR, C-reactive protein, and additionally adjusting for kidney function with GFR (mGFR, eGFRcrea, and eGFRcys). as all these variables are confounding the relationship between NO-precursors or DMA and CVD.

Figure S2. Study population selection in Renal Iohexol Clearance Survey (RENIS).


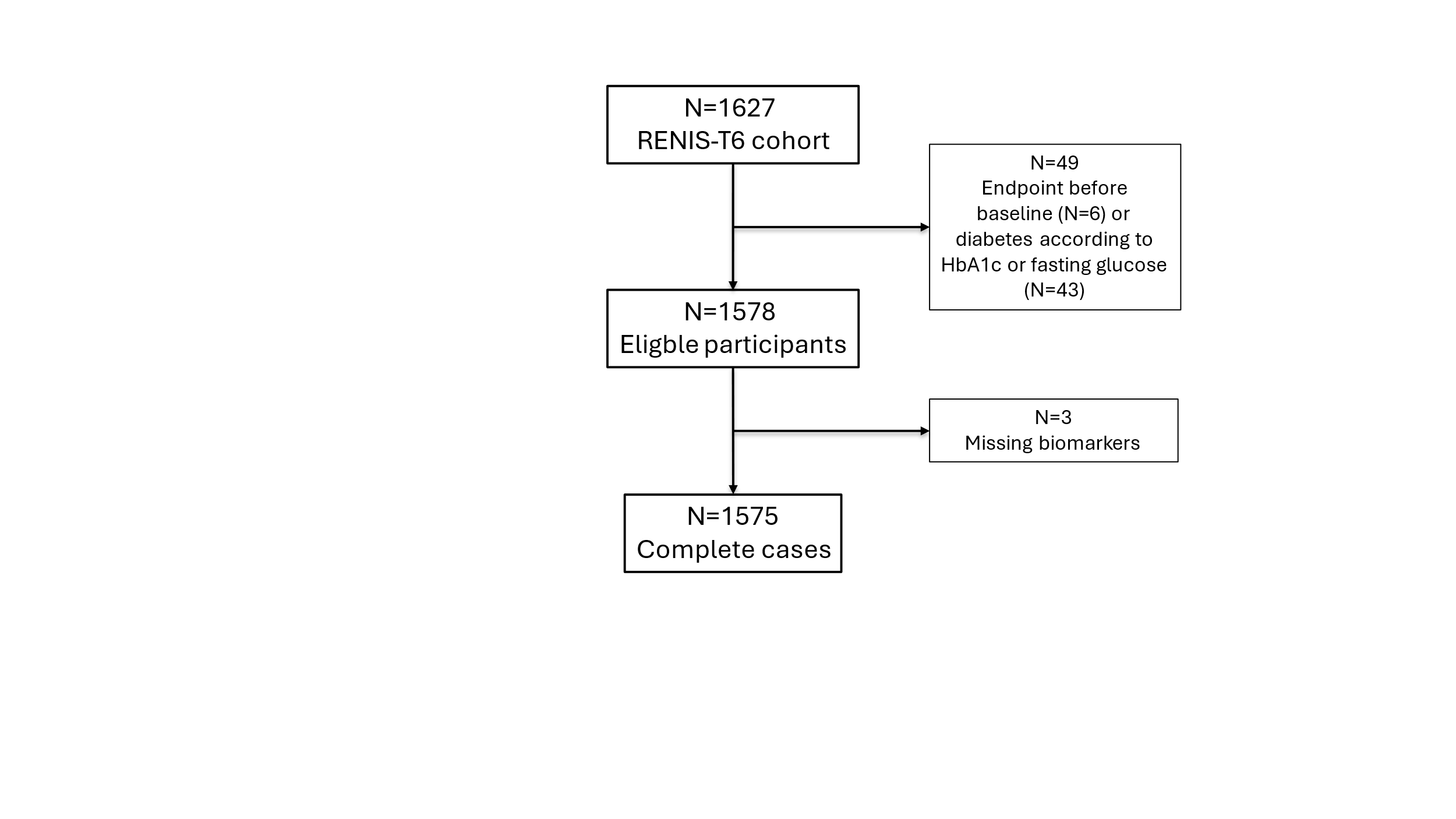


Figure S3: Histogram of study participants distributed on biomarkers concentration: ADMA (a), SDMA (b), arginine (c), citrulline (d), and ornithine (e).

|  |  |
| --- | --- |
|  |  |
|  |  |

1. MORGAM Project MM. MORGRAM Project e-publications: National Institute for Health and Welfare and the MORGAM Project investigators; 2001 [updated 06.05.2024; cited 2024 25.09.2024]. URN:NBN:fi-fe20041529 [Available from: <https://www.thl.fi/publications/morgam/manual/followup/form22.htm>
